# Anterior Matrix Systems for Composite Restorations: A Review

**DOI:** 10.7759/cureus.37145

**Published:** 2023-04-04

**Authors:** Neha K Urkande, Nikhil Mankar, Pradnya P Nikhade, Manoj Chandak, Anuja Ikhar, Aditya Patel

**Affiliations:** 1 Department of Conservative Dentistry and Endodontics, Sharad Pawar Dental College and Hospital, Datta Meghe Institute of Medical Sciences, Wardha, IND

**Keywords:** wedges, proximal contact, retainer, band, matrix

## Abstract

In the field of restorative dentistry, composite resins have emerged as the most utilized materials. They revolutionized the cosmetic approach to anterior tooth treatment since their introduction to dental practice. In cases where one or more of the tooth's walls are absent, matrices are employed to assist in the creation of the proper anatomic contour and proximal contacts without the occurrence of gingival overhangs. Matrices come in a variety of designs suitable for various therapeutic conditions. Depending on the level of tooth destruction, they might range from a simple metal or plastic strip to a circumferential ring of metal surrounding the entire crown. Although the handling characteristics of modern composites have significantly improved, dentists still encounter challenges with the development of good interproximal contact and proper anatomical contour. The utilization of an anterior matrix system can be paramount in achieving these objectives.

## Introduction and background

Successful placement of direct composite restorations, particularly in the aesthetic zone, requires a comprehensive understanding of dental anatomy, including knowledge of color and material science. Achieving proper contour and tight proximal contacts represents one of the most challenging aspects of this procedure [1]. Various approaches can assist the dentist in correctly restoring a tight, well-contoured proximal contact surface. Various matrices and approaches have been documented to aid practitioners in producing predictable results, such as the use of a silicone index alone or in combination with Teflon tape, through Mylar strip, anterior transparent matrix, and anterior/posterior metallic matrices [2]. Because of their simplicity and versatility, real anatomical matrix systems, as well as the injectable molding technique and others, have lately brought aesthetic restorations within everyone's reach, allowing the dentist to capture the final form and volume of the material utilized. The restoration of a functionally and morphologically correct tooth shape and proximal contact areas (PCAs) allows the formation of harmonious interdental papillae, prevents food lodgment, and aids in the stabilization of the dental arches by maintaining all the teeth in positive contact with each other [2].

The words contact point and contact area have been used interchangeably in the literature. According to research, PCAs rather than contact points are detected between maxillary anterior teeth. When contacting surfaces have ideal curvatures, contact points appear, which is common in young patients with newly erupted teeth. When repairing teeth in a clinical context, the size of anterior PCAs should be considered [3]. The anatomical shape of the teeth (rectangular, triangular, oval), the kind (canines, laterals, central incisors), the surface location (lateral, mesial), and the distance between the teeth all influence the shape of each contact region of maxillary anterior teeth (diastemas, crowding, loss of interproximal space due to poor restorations). Due to the vast number of initial requirements that must be met, having a large number of anatomically prepared matrices at one's disposal is necessary [3]. This article provides the use of various anterior matrix systems used while restoring composite restoration, particularly in the aesthetic zone.

## Review

Benefits of an ideal contact and contour

Ideal contact and contour maintains periodontal health, prevents food lodgement, provides support, alignment, and stabilization, self-cleansing, the shelf life of restorations is improved, and maintains the teeth's normal mesiodistal relationship [4].

Matricing and Matrices

Matricing is the process of constructing a temporary wall that runs in the opposite direction of the axial wall to surround the lost tooth structure [4]. "Matrix" is a tool that holds the restoration in place in the tooth while it sets [4]. The word matrix is derived from the Latin word "Mater" which means "Mother." It was introduced in the year 1871 by Dr. Louis Jack. The matrix is a device used to contour a restoration to simulate that of a tooth structure, which it is replacing [5].

Ideal Requirement

It should have ease of application, ease of removal, provide proper proximal contact and contour, and should be cost-effective and confine the restoration [4,5].

Dental matrix systems: historical development

**Figure 1:**
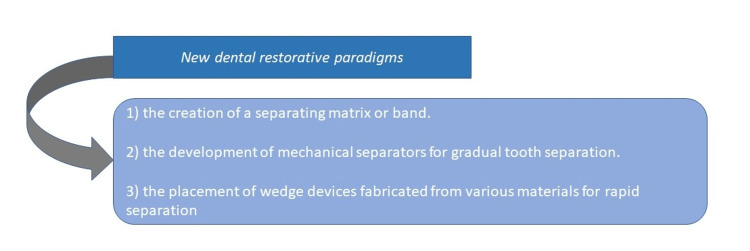
New dental restorative paradigms

Modern dentistry should prioritize a new approach to restorative care that involves preserving tooth structure with minimal intervention, optimizing tooth form and function, and improving proficiency in aesthetic technology and material sciences [6]. During mastication, deglutition, and phonetics, continuous intraoral centric and eccentric functional movements cause constant transposition of dentition, leading to increased attritional forces and changes in proximal contact surface positions [6]. Proper interproximal contour, optimal proximal contact surfaces, constant marginal ridge elevations, and central groove continuity within an arch are crucial for maintaining a healthy oral cavity [7]. The selection of dental materials and methods should be based on the etiology to achieve effective rehabilitative procedures that incorporate these aspects. Historically, the concept of form and function, including the proper shape of proximal surfaces, was developed in the 19th century [8]. Contoured restorations, as shown in Figure 1, promote the creation of normal contact surfaces, thereby supporting a healthy tooth periodontal complex.

The technique of modern operative dentistry using filling materials was effectively adopted with these components in place. Finally, the restoration of natural tooth structure and function was accomplished [8].

Classification**

**Figure 2:**
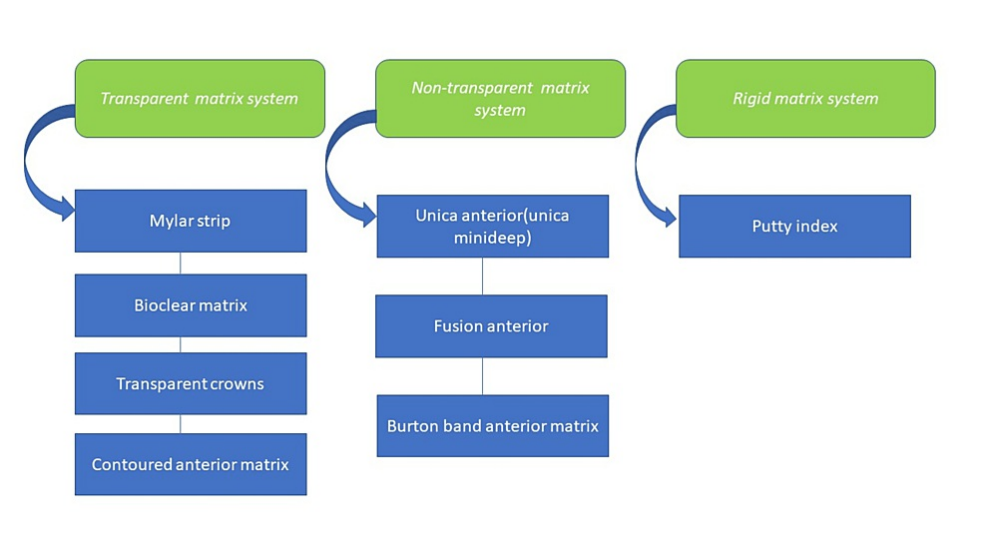
Classification of the anterior matrix system

The newer classification (Figure 2) is based on need: transparent matrix system, non-transparent matrix system, and rigid matrix system. Except for faults that are smaller where a flexible Mylar strip will do, they satisfactorily restore suitable contour and contact form. Additionally, flaws that extend from the labial to the palatal surfaces will necessitate a stiffer putty index.

Teflon tape 

Clear Teflon and cellulose acetate strips are examples of typical interproximal matrices. These strips are useful because the resin can be photoactivated through the transparent matrix. When stretched, polytetrafluoroethylene (PTFE) tape can act as a thin interdental barrier and create a restorative contact area that is reasonably accurate. A wedge or flat plastic instrument can be used to momentarily separate the teeth to make it easier to put the PTFE tape between difficult-to-navigate connections. To ensure close adaptation to the dry tooth surface, folds or creases that may arise during implantation and stretching via the interdental contact area can be removed with the aid of a microbrush or sable-hair brush [9]. Applying PTFE tape will help ensure proper positioning and seating because it won't affect an indirect adhesive restoration's seating or a tooth mold index. Because of these characteristics, it can be used as a separating medium when placing composite restorations, whether free-hand, using a tooth mold index, or when cementing adhesive restorations [10].

Mylar strip

When the adjacent tooth has a flat contact region, the Mylar strip can be applied using a pull-through technique. When employed alone, this matrix's flexibility makes it difficult to contour vast areas, which results in uneven connections and contours. The matrix's stabilization during the restoration of nearby lesions is another frequent issue. Multiple surfaces of the same tooth and numerous adjacent lesions must be restored, which is time-consuming and difficult. None of these matrices can be employed effectively while treating Class V cervical defects as well [11].

Modified putty index using Mylar strip

A putty index of the incisors is made when the defect to be corrected in incisors teeth is built up using direct technique or indirect technique. The index is created using either addition or condensation silicone putty materials, where the addition silicone putty material is the favored material.

The putty index is placed on the palatal surface for composite insertion after acid-etching and bonding agent application on the tooth surface to be repaired. At this stage, a Mylar strip is placed over the adjacent tooth to prevent the adhesion of composite material, which differs from the typical approach [12]. With a putty index alone or in combination with a flexible matrix like PTFE (Teflon) tape or a Mylar strip, composite resin restorations have been placed in anterior teeth. Around the teeth and past the contact area, the flexible matrix is challenging to manipulate [12].

**Figure 3:**
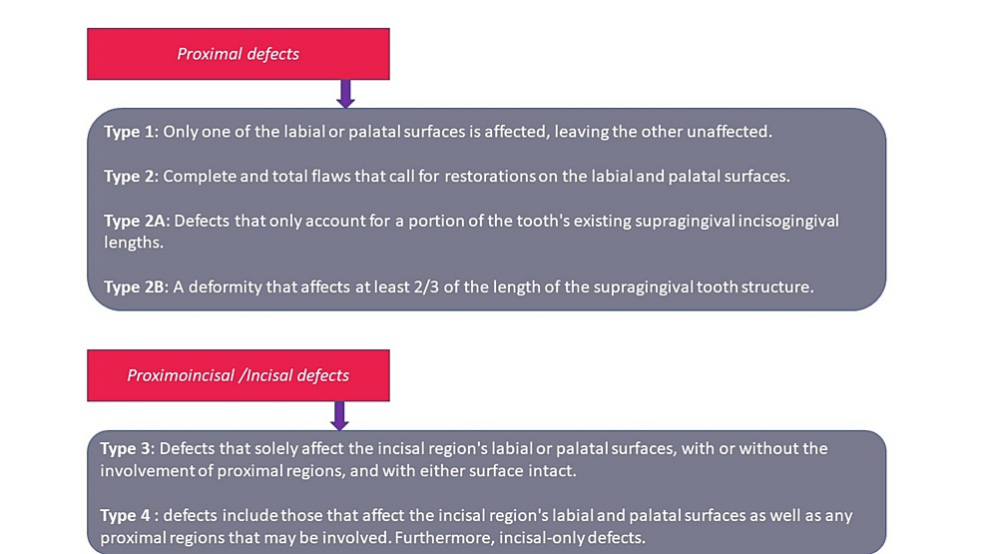
Classification of proximal defects of anterior teeth

**Figure 4:**
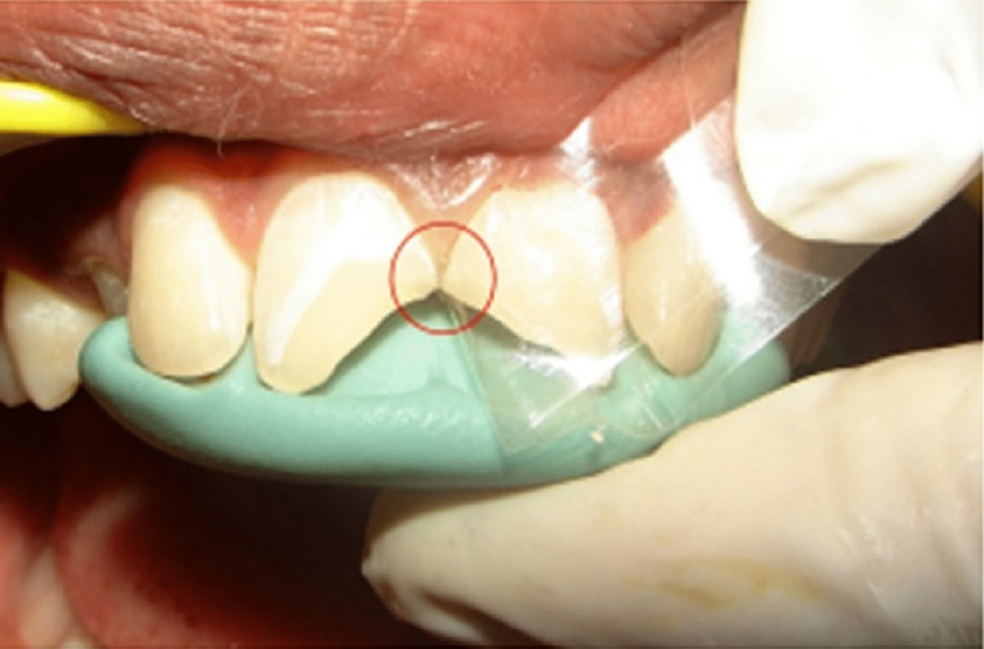
Mylar strip and putty index in patient's tooth This is an open-access image that adheres to the stipulations set forth by the Creative Commons Attribution License, as implemented by John Wiley & Sons Ltd.

**Figure 5:**
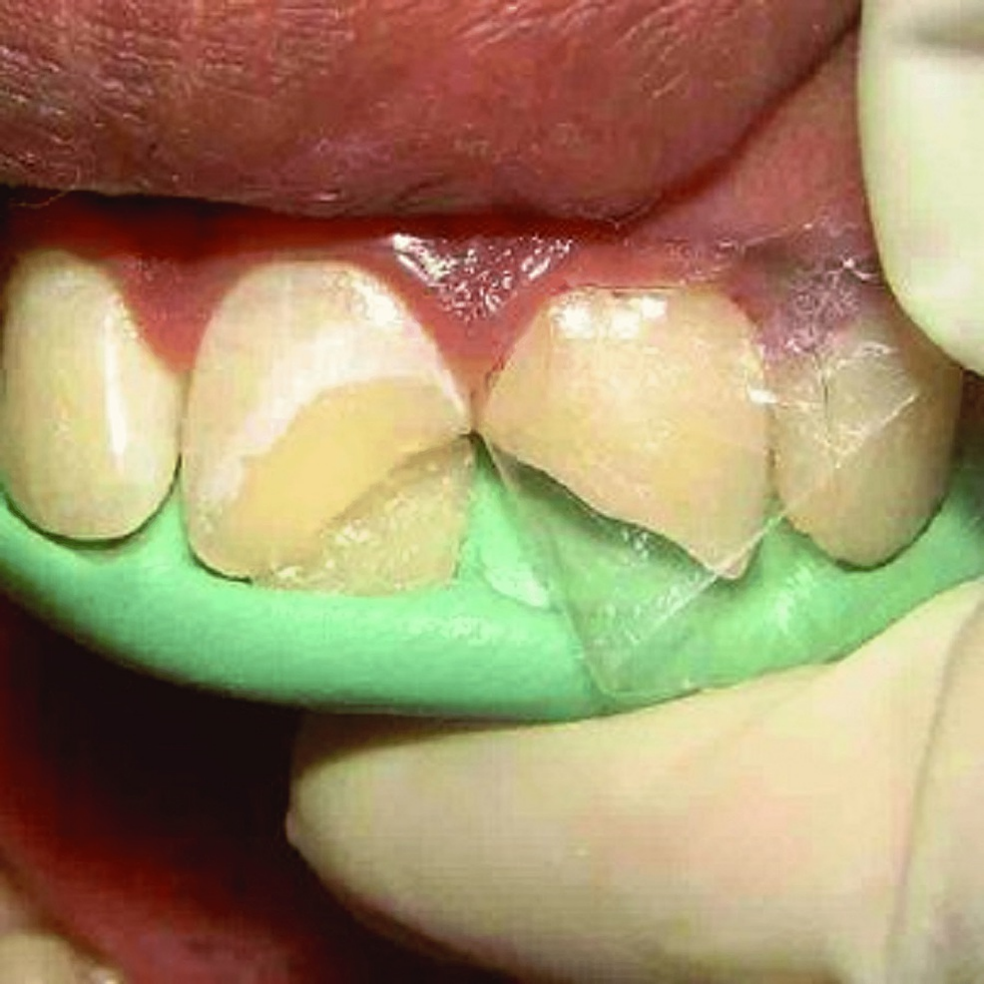
Palatal shelf build using Mylar strip and putty index This is an open-access image under the terms of the Creative Commons Attribution License by John Wiley & Sons Ltd.

An alternative is to use a clear silicone index. However, because silicone is sticky, it might be challenging to construct, with which several direct composite resin restorations can be placed successfully. It enables the polymerization of the composite resin through the transparent silicone material as well as the transfer of the tooth morphology from the waxed cast to the mouth. The index is made stiff by the putty, and the usage of composite resins that light-polymerize is made possible by the clear silicone. In the front or back of the mouth, this method can be used to repair a single tooth or a row of teeth [13-15]. This more recent classification given by Dr. G.V. Black (Figure 3) is based on the fact that larger proximal defects will require a stronger and better supporting putty index matrix to satisfactorily restore acceptable contour and contact form, as opposed to smaller defects, where a flexible Mylar strip will be sufficient (Figure 4). Additionally, defects that will extend from the labial to the palatal surfaces (Figure 5) will necessitate a stiffer putty index (through and through defects) [16-19].

Advantages 

When a rigid matrix is utilized to restore the palatal surface, it creates the correct contour and length of the incisal edge, which can then be used to guide and support the labial surface composite buildup. Because of the adaptability of this approach, it can also help with palatal surface moisture control. When used in the labial surface buildup, flexible matrix Mylar strips help achieve the ideal aesthetic anatomic contour and outstanding labial surface finish. It can be employed in challenging circumstances like multiple tooth restorations, crowded teeth, and severely defective restorations [20-22].

Limitations 

It has been observed that restorations may necessitate a second appointment for the patient to insert both the Mylar strip and the index together during the initial phases of use. This technique will also require the use of four hands, as the operator will be required to manipulate and position both the Mylar strip and the putty index concurrently [23].

Disadvantages 

Flexibility, especially in major defect restorations, can contribute to incorrect contour and contact establishment, unable to achieve exact contour in the palatal aspect of restorations [24].

Transparent crown

For more than three decades, damaged and carious anterior teeth have been restored using the cellulose transparent crown (also called a strip crown). Their great durability and retention performance when used to produce bonded resin composite restorations in primary incisors have been documented in the literature. These cheap and efficient crown formers can be utilized in pediatric dentistry, where they have been found to have a success rate of over 80%, but they can also be used, when necessary, in permanent dentition for patients of all ages. Many of the pre-formed sizes are substantially larger than the normal primary incisor and more closely resemble the size of the permanent dentition [25]. When tooth tissue has been lost from many surfaces, generally as a result of cavities or trauma, they are traditionally used for primary full coronal restorations. They are frequently employed in the development of microdont incisors as well. They can offer a relatively simple method to obtain a greater aesthetic outcome, which can then be quickly fixed if they break down again in the future [26].

Bioclear matrix system

Bioclear was introduced in the year 2007 by Dr. David Clark [27]. Anterior matrices from bioclear allow for a more current approach to composite dentistry. They are utilized for restorative dentistry and aesthetic procedures where small areas need to be filled and have less curvature than diastema closure matrices. The anatomic structure of the bioclear matrix allows for predictable repair or changes to a tooth's emerging profile. The matrix can be utilized wedge-free to close narrow areas with a large contact. The papilla stabilizes and minimally seals the matrix once it is put in the sulcus. The anatomically formed matrices from bioclear allow the composite to be injected/placed into the embrasure without the risk of an overhanging margin. The bioclear matrix smooths up and contours the interproximal composite [27].

Features

They have excellent cervical adaptation, superior to flat Mylar strips, preserve the gingival papilla, have easy matrix selection, and each matrix's incisal tab indicates the proper orientation.

Indications for Use

All anterior cases can be treated with the bioclear anterior matrix kit. Bioclear standard anterior matrices are used in both routine restorative dentistry and aesthetic procedures to develop new and exaggerated emergence profiles. Diastema closure matrices are employed as well to close diastema more than 1 mm and to close big black triangles [28].

Unica anterior

It is developed by Polydentia in partnership with Style Italiano.

Indications 

Unica anterior matrix system is indicated in Class III, IV, and V anterior restorations and direct composite veneers.

Features 

Unica anterior is an easy and suitable matrix and is cost-effective. Unica anterior's curved shape responds to the various morphologies of anterior teeth and allows for simultaneous restoration of the proximal and cervical margins, even using rubber dams and/or gingival retraction cords, considerably reducing the chair time. The placement wings on the matrix enable quick and efficient positioning [28]. Unica anterior allows restoration of both proximal and cervical margins at once. It anatomically restores the proximal margins to their contoured profile and easily manages the cervical area to the predictability of the restoration and gingival retraction.

Unica minideep**

It is particularly made using a malleable alloy that may take on the necessary shape for smaller anterior teeth. The best matrix for aesthetic restorations such as direct stratification composite veneers and shaping changes to maxillary central incisors, as well as direct Class III, IV, and V [28].

Indication 

Unica minideep is indicated for maxillary and mandibular lateral incisors, mandibular central incisors, conoid teeth, triangular teeth, peg laterals, and teeth with narrow cervical diameters.

Fusion^TM^ Anterior Matrix System

The Fusion^TM^ Anterior Matrix System was introduced by Garrison Dental.

Indications 

An anterior matrix system is indicated in anterior restorations like Class III and IV crowns and composite veneers. The robust stainless steel matrix slips effortlessly into the sulcus, keeping its shape and contour deprived of distortion. The appropriate anatomical curvature is produced in a gingivo - incisal and facial - lingual orientation when properly inserted. The Fusion Anterior Wedge is utilized to help preserve this ideal anatomical position by ensuring a tight seal at the cervical third from facial to lingual. These drastically curved wedges let you focus on composite placement and simplify the restorative procedure [29]. 0.0015” thick, firm stainless steel resists deformation during placement, ease of placement in case of deep restoration, ideal anatomic curvature in the gingivo-incisal and facial-lingual direction, and superior for restoring deep carious lesions.

Features

It has a firm seal at the cervical margin, maintains the ideal anatomy of a tooth, and the unique "T" design rests deep in the interproximal region reducing the feared "black triangle." The cervical interface provides better adaptation with deep restorations. For deeper restorations, the strong metal matrix bands are substantially thinner than typical plastic strips and can be put through existing contacts and into the sulcus. Ideal gingival/incisal anatomy and facial/lingual anatomy are pre-built, making sculpting in the crucial anterior area more easier.

The Fusion Anterior wedge's distinctive, extreme curvature tightly wraps the band around the tooth and holds it in place, freeing up the clinician's hands. They smoothly slide alongside the interdental papilla to sit lower interproximally, allowing for maximum tooth separation while also helping to prevent black triangles [29].

*Refill Bands* 

The two types of refill bands are AN-100 Fusion^TM^ Anterior Matrix Bands Short, 8.6 mm, and AN-200 Fusion^TM^ Anterior Matrix Bands High, 10.4 mm.

Burtonbands anterior matrix system

It is designed by Dr. Matthew Burton. An accurate contour and embrasure placement are essential for a natural-looking anterior repair. The proximal wall is helped to develop by the anterior matrix system of the Burtonbands. In an otherwise extremely changing environment, the Burtonbands system offers a constant, resulting in more reliable and effective repair [30].

Design

A 38-micron metal matrix is affixed to a plastic wedge in the design. The wedge's notched edge clicks into position, stabilizing the matrix and releasing both hands for restoration. The wedge has a distinctive handle form, and the matrix is curved yet flexible, allowing it to follow the shape of the tooth from the root surface to the incisal edge. You can use cotton tweezers or your fingers to position the square head. No specialized equipment is needed.

Benefits 

It allows for complete access to the restoration, improving the face embrasures formation and enabling the incisal embrasure to be shaped and positioned correctly. It also enables simple installation in a single step without requiring significant matrix adjustment. Control over contour and proximal contact is possible due to the metal matrix's burnishability and a narrower profile than plastic strips. It enhances the establishment of proximal contact by introducing a little space between the teeth. Consistently seal the gingival margin to prevent isolation problems, extra flash, and overhang margins, while allowing access to subgingival cavities that are difficult to reach. The height of the matrix can be reduced for diastema closure scenarios [30].

Blue View VariStrip^TM ^(contoured anterior matrix)

The Blue View VariStrip^TM^ is a new contoured anterior matrix. It provides the ideal curvature and band height for practically all anterior restorations. From one end to the other, the 0.05 mm thin (plastic) anatomical strip is tapered. The strip can be placed interproximally and then slid until the tooth heights are exactly aligned. The occluso-gingival anatomy is easily recreated with pre-contouring, and flat embrasures are avoided [30]. Blue tint adds contrasts between the matrix and the tooth structures without jeopardizing composite resin polymerization. Variable height from 5 to 10 mm ensures that all anterior teeth are at the proper height. Straight Mylars can sometimes produce inaccurate flat embrasures, black triangles, and food entraps. It is excellent for Class IV and diastema closures. Blue tint does not affect the cure but ends the annoyance of losing clear strips [30].

## Conclusions

Achieving physiologic tight contact is a significant challenge while restoring anterior teeth. Preventing gingival overhang improves proper occlusion and periodontal health. The process of matricing is essential while placing restorations. There are numerous matrix systems on the market right now. The best matrix for a specific clinical situation should be used by the clinician. To provide the best contacts and contours, the matrix should be chosen based on how simple it is to use and how effective it is. The individualized matrix technique offers a method to accurately produce controlled, functional, esthetic, and long-lasting outcomes.
